# Role of glucocorticoid receptor mutations in hypertension and adrenal gland hyperplasia

**DOI:** 10.1007/s00424-022-02715-6

**Published:** 2022-06-22

**Authors:** Sophia Verouti, Edith Hummler, Paul-Emmanuel Vanderriele

**Affiliations:** 1 Department of Nephrology, Hypertension and Clinical Pharmacology, University of Bern, CH-3010 Bern, Switzerland; 2 National Center of Competence in Research, Kidney.CH, CH-1011 Lausanne, Switzerland; 3 Department of Biomedical Sciences, University of Lausanne, Rue du Bugnon 27, CH-1011 Lausanne, Switzerland

**Keywords:** Glucocorticoid resistance, Hypercortisolism, Homeostasis, Kidney physiology, Epithelial transport, Animal model

## Abstract

Hypertension is one of the leading causes of premature death in humans and exhibits a complex aetiology including environmental and genetic factors. Mutations within the glucocorticoid receptor (GR) can cause glucocorticoid resistance, which is characterized by several clinical features like hypercortisolism, hypokalaemia, adrenal hyperplasia and hypertension. Altered glucocorticoid receptor signalling further affects sodium and potassium homeostasis as well as blood pressure regulation and cell proliferation and differentiation that influence organ development and function. In salt-sensitive hypertension, excessive renal salt transport and sympathetic nervous system stimulation may occur simultaneously, and, thus, both the mineralocorticoid receptor (MR) and the GR-signalling may be implicated or even act interdependently. This review focuses on identified GR mutations in human primary generalized glucocorticoid resistance (PGGR) patients and their related clinical phenotype with specific emphasis on adrenal gland hyperplasia and hypertension. We compare these findings to mouse and rat mutants harbouring genetically engineered mutations to further dissect the cause and/or the consequence of clinical features which are common or different.

## Introduction

Hypertension is a multifactorial disease and affects one-third of the human adult population [36]. It has been recently identified as the most prevalent cardiovascular comorbidity in patients infected with COVID-19 significantly increasing the risk of hospitalization and death [40, 53]. Glucocorticoid resistance is a condition characterized by generalized, partial target tissue resistance to this hormone [48]. Compensatory mechanisms lead to elevation in circulating adrenal steroids with mineralocorticoid and/or androgenic activity, and the clinical spectrum of this condition is broad, ranging from asymptomatic to severe cases of hyperandrogenism, fatigue and/or mineralocorticoid excess. The molecular basis of glucocorticoid resistance has been attributed to mutations in the GR gene impairing glucocorticoid signal transduction [2]. The role of the glucocorticoid receptor in salt-sensitive hypertension is less understood in human, and, therefore, animal models carrying GR mutations are useful to study the cause and the development of this disease.

**Fig. 1:**
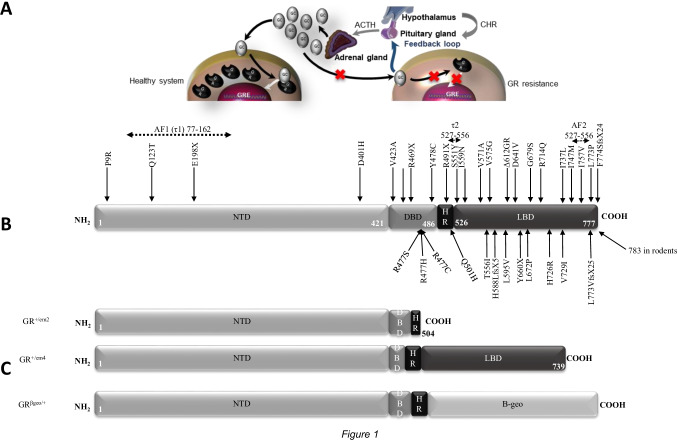
Pathogenesis of glucocorticoid resistance. **A** Consequences of GR mutations on the hypothalamic–pituitary axis causing hypercortisolism. ACTH adenocorticotropic hormone; CRH corticotropin-releasing hormone **B** Linear model of the human GR structure and localization of identified mutations. **C** Linear structure of rodent GRs carrying the mutant em2, em4 and β geo allele; NTD N-terminus domain, DBD DNA binding domain, HR hinge region, LBD ligand binding domain

Glucocorticoids are ligands of the glucocorticoid receptor (GR), which is one of the most studied transcriptional regulatory factors (TRFs) involved in homeostasis and physiological regulation [76]. Glucocorticoids are small lipophilic signalling molecules produced by the adrenocortex under tight regulation of the hypothalamic–pituitary–adrenal gland (HPA) axis [63] (Fig. 1A). They present fundamental hormones playing a role in various biological functions as metabolism, inflammation processes or stress through the control of more than 1000 genes [20]. These hormones are released rhythmically with both a circadian and an ultradian (pulsatile) pattern [20]. Despite a precise regulation, glucocorticoids are sometimes secreted in excess or in an insufficient manner [57]. GR mutations leading to glucocorticoid insensitivity can affect glucocorticoid production through a dysregulation of the feedback loop (Fig. 1A) leading to a large spectrum of clinical phenotypes as adrenal hyperplasia and hypertension. Sustained glucocorticoid excess together with high salt intake might induce salt-sensitive hypertension in human, but it was not reported yet whether varying salt intake differentially affected subjects carrying GR mutations. Studies in animal models associated glucocorticoid excess with ACTH-induced steroidogenesis, increased deoxycorticosterone levels and/or increased glucocorticoid-mediated MR signalling, but also with abnormal renal hemodynamic response and failure of the distal nephron to adapt to high salt intake [26].

In human, there are still questions that need to be addressed, (i) if and which kind of GR mutations are causative for the development of GR-mediated hypertension, and (ii) which are the main predisposing factors for this disease. Moreover, it was not clear until recently if some of these clinical features might be recapitulated in animal models to help dissect the underlying molecular mechanism. The aim of this review is to give an overview of GR pathological mutations found in humans and on studies of GR mutations done in experimental animals. We will discuss the prevalence of hypertension and adrenal hyperplasia in patients and animals carrying GR mutations and discuss possible mechanisms.

### Primary structure of the glucocorticoid receptor

The primary structure of the human glucocorticoid receptor is known since 1985 [45], which is the date of its first reported cloning. The nuclear receptor subfamily 3 group C member 1 (*NR3C1*) gene, located on the short arm of chromosome 5 (5q31Y32) [14], encodes the GR. This gene is composed of nine exons (1 noncoding and 8 coding exons). Alternative splicing produces two main protein isoforms, namely hGRα and hGRβ leading further to eight receptor α and eight β isoforms [50]. The two isoforms are similar up to position 727, and hGRα is longer of 50 amino acids [52]. In 1990, Zong and colleagues [24] demonstrated that the promoter region (5′UTR-untranslated region) of human GR has a GC content of 72% but does not contain a TATA or CAAT box. The 184 nucleotides of exon 1 represent only the 5′UTR and are not coding for the protein. The major transcription start site is situated in exon 2, 134 bp upstream of the ATG initiation codon [23].

The 5′ part of the sequence encodes the N-terminus domain (NTD, 421 amino acids) of the receptor (Fig. 1). The main component of the NTD is the ligand-independent constitutive transcriptional activation function 1 (AF1), also named tau1 (Fig. 1B). AF1 is rich in acidic amino acids and necessary for GR transcriptional activation and for the interaction of GR with co-regulators, chromatin modulators and the basal transcription machinery. The NTD is followed by the DNA binding domain (DBD). A particularity of the DBD is the two highly conserved zinc fingers which are required for the DNA binding specificity of the receptor and the dimerization. These subdomains contain four cysteine residues coordinating a zinc ion, followed by an amphipathic helix and a peptide loop [76]. The loop of the second zinc finger is important for the dimerization via the distal box (D box) [76]. The DBD is the most conserved region among the nuclear hormone receptors, whereas the NTD is the most variable domain [34]. Between DBD and the ligand-binding domain (LBD), a small and flexible region is present from amino-acid 488 to amino-acid 527, the hinge region (HR) (Fig. 1B). Recent studies demonstrated its role in circadian transcriptional activity of the GR [32, 44]. At the C-terminus of the GR is the LBD encoded by exons 5 to 9. The LBD comprises three important regions, the transactivating domain 2 (Tau2), AF2 necessary for ligand-dependent interactions with co-regulators and an additional nuclear localization signal (NLS) (Fig. 1B). The hGR is virtually expressed in all tissues [69].

### NR3C1 gene mutations in humans

Since 1982, 38 *NR3C1* gene mutations have been described in human (summarized in Fig. 1; Table 1). Among these mutations, four have been described separately in unrelated patients (G679S, R477S, L672P and R714Q). G679S is found both as homo- and heterozygous mutation in human. In 2001, Raef et al. [56] demonstrated that the homozygous G679S mutation of the GR-α gene is associated with severe cortisol resistance, whereas a heterozygous mutation can lead to subclinical cortisol resistance. The R477S mutation presents different clinical phenotypes (adrenal hyperplasia vs no adrenal hyperplasia; normotensive vs hypertensive). In total, 38 GR mutations are listed in Table 1, with their related clinical phenotype documented at the age of diagnosis as well as in follow-up studies. For example, the E198X mutation has been discovered in a 3-year-old patient without adrenal hyperplasia [64]. However, at the age of 12 years, this patient additionally presented with bilateral adrenal hyperplasia [64]. Overall, the age of diagnosed patients is ranging from 5 weeks to 70 years with an average of 32.8 ± 19 years even though, in families with GR mutations, the age of affected subjects is not always mentioned. Over the past years, the number of diagnosed patients carrying GR mutations is continuously increasing, and 16 hitherto unknown GR mutations were documented within the last 6 years. Overall, 26 new mutations were identified in women versus 16 in men (Table 1). It might be interesting to follow whether there is a generally higher incidence in women. The large majority are heterozygous (31 cases) in comparison to six homozygous GR mutations. Homozygous mutations might be underrepresented due to the severity of the clinical symptoms.

**Table 1:**
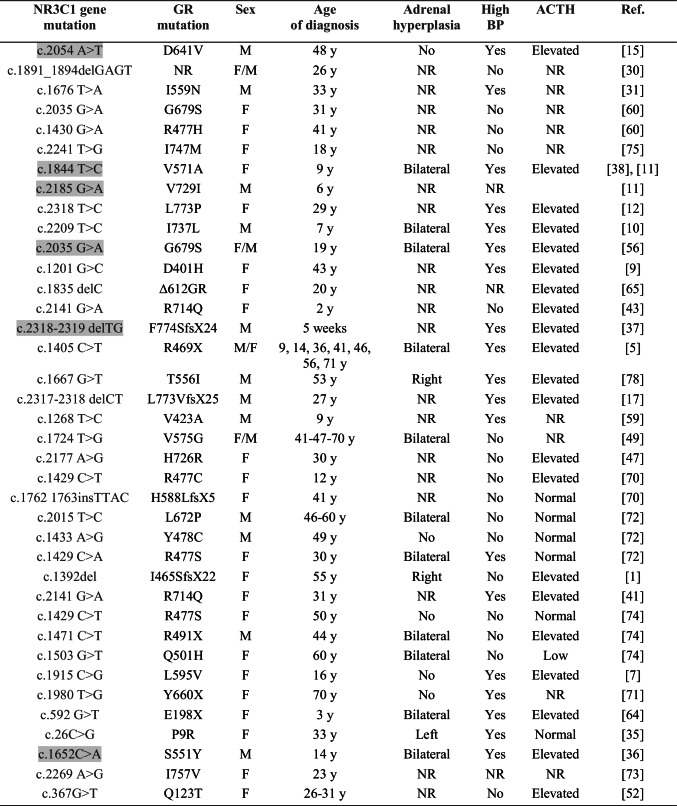
Overview on documented NR3C1 mutations with their phenotype

Homozygous mutations are indicated in grey

*NR* not reported,* F* female, *M* male, *y* year, *BP* blood pressure, *ACTH* adrenocorticotropic hormone

### Molecular features of the NR3C1 gene mutations

In human, 62% of the *NR3C1* gene mutations affect the LBD (Fig. 1), and, despite its size and its variability in the nuclear receptor family, only four mutations are localized within the N-terminus domain, with two of these situated within the AF1 domain (Q123T and E198X). Tatsi et al. [64] showed that E198X was a nonsense mutation resulting in a premature stop. The loss of 577 amino acids leads to degradation through the nonsense-mediated mRNA decay (NMD) mechanism. Q123T leads to glucocorticoid resistance and is characterized by a truncated non-functional protein [52]. The mutant D401H equally results in a truncated protein [9], whereas the P9R variant presents a missense mutation [35]. Two mutations (S551Y and T556I) are localized in the tau 2 domain of the LBD and I757V in the AF2 domain, respectively [73] (Fig. 1; Table 1). The homozygous S551Y mutation did not affect GR protein abundance but decreased its transcriptional activity, nuclear translocation and dimerization [36]. The T556I mutation did not affect GR protein abundance [78].

In vitro studies using transfections allowed to quantify the fluorescence of the different GR mutants in distinct subcellular compartments and to determine GR-dependent nuclear translocation [72]. The Δ612GR mutant GR protein could not be detected [65]. In truncated GR mutants Q123T, E198X and R469X, protein abundance is decreased [5, 52, 64]. Ligand binding is normal in D401H, V423A, R477H, R477S and Y478C mutations and decreased in S551Y, T556I, I559N, V571A, V575G, D641V, G679S, R714Q, H726R, V729I, I737L, I747M and L773VfsX25 mutations and lacking in the Δ612GR, L672P and F774SfsX24 mutations. Data are not reported for six mutations (H588LfsX5, L595V, P9R, R477C, I757V and I465SfsX22: Table 1). DNA binding is preserved in D401H, T556I, V575G, G679S, R714Q, H726R and I737L mutations, decreased in 2 mutations (V423A and Y478C; [59, 72]) and lacking in 3 mutations (R477H, R477S and L672P; [60, 72, 74]). Transactivation is only increased in the D401H mutation and lacks in the R477S, R491X, F774SfsX24, Δ612GR, Y660X and L672P mutations. Translocation of the GR-glucocorticoid complex is diminished in most of the GR mutations. Only the D401H mutation presents normal trafficking [9], and Δ612GR and L672P mutations depict no translocation of the GR-glucocorticoid complex [65, 72]. Overall, most described GR mutations exhibit a normal protein abundance, but decreased ligand binding, transactivation and translocation.

### Clinical symptoms of NR3C1 mutation

**Fig. 2:**
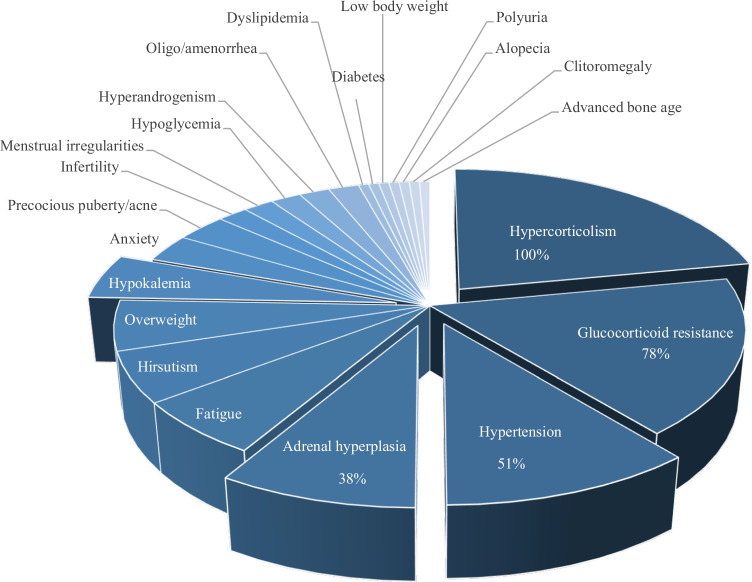
Graphic representing percentages of all clinical features observed in patients carrying 38 GR mutations (see Table 1) causing glucocorticoid resistance

Glucocorticoids are key hormones involved in multiple pathways and physiological processes as development, metabolism, or immunity [73]. Any GR mutation thus affects multiple pathways and might thus affect organ development and function leading to a systemic phenotype (Fig. 2). Patients carrying heterozygous or homozygous GR mutations present with hypercortisolism (100%), hypertension (51.3%), adrenal hyperplasia (38%), fatigue (28%), anxiety, hirsutism (25%) and hypokalaemia (21.6%: Fig. 2). Irregular menstruation cycle, infertility, obesity or low body weight, dyslipidaemia, diabetes, hyperandrogenism, polyuria, ambiguous genitalia, oligo/amenorrhea, hypoglycaemia, alopecia, acne and precocious pubarche, clitoromegaly and advanced bone age symptoms are more rare [52, 73] (Fig. 2). A few human studies additionally suggested impairment of sodium homeostasis although plasma Na^+^ levels were in the normal range [33, 36]. To further answer this question, more detailed and standardized phenotyping might be required in the future. More recently, Vitellius and Lombes concluded that there is no clear genotype–phenotype relationship between the severity of the disease and the degree of functional loss of the mutant GR and the age at presentation. On the other side, there was a tendency that young patients presented more severe clinical signs than adults [73]. The reason is, however, not known, and more case studies might be required. In the following part, we will further discuss the link between GR mutation, adrenal gland hyperplasia and hypertension.

### Is adrenal hyperplasia a symptom of GR resistance?

In 2018, Vitellius et al. hypothesized that GR mutations might represent a novel genetic cause of adrenal hyperplasia [74]. In the French MUTA-GR study including 100 patients, they discovered 2 new mutations (R491X, Q501H) and 3 already described GR mutations (R469X, R477S and L672P). The prevalence of patients exhibiting adrenal hyperplasia and hypertension and/or presenting hypercortisolism is 5% thus encouraging further GR mutation screening to differentiate GR resistance from Cushing’s syndromes and to improve the follow-up of these patients. It is worthwhile mentioning that not all cases of PGGR are attributed to *NR3C1*gene mutations [46]. Among the 38 GR mutations identified in human, 14 (38%) are associated with adrenal gland hyperplasia (Table 1). Seven of these mutations are present in the LBD, 3 in the DBD (L672P, R477S and R469X), 2 in the HR (I465SfsX22 and R491X) and 2 in the NTD (P9R, E198X). The adrenal hyperplasia is significantly bilateral (11 cases versus 3 cases unilateral: Table 1). The relatively high percentage of adrenal hyperplasia strongly suggests the GR mutations being causative. Furthermore, a recent study done in 389 patients determining the genetic predisposition to primary bilateral macronodular adrenal hyperplasia (PBMAH) found a prevalence of 8.9% GR mutations [67].

**Fig. 3:**
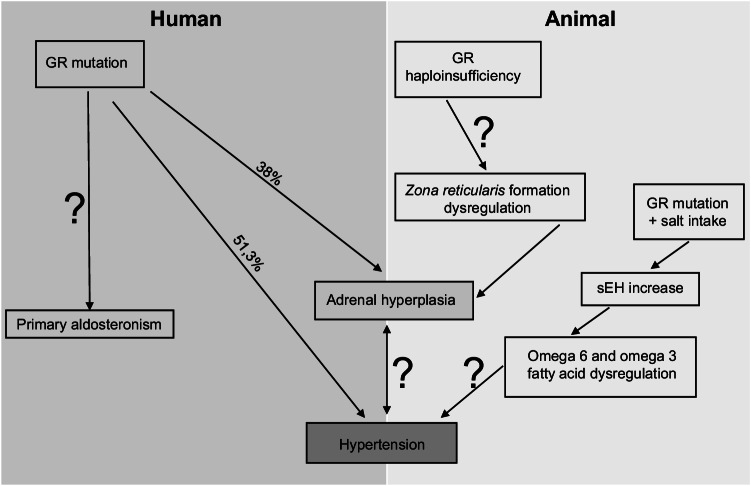
Hypothetical scheme of mechanisms implicated in the generation of salt -sensitive hypertension and adrenal hyperplasia in human and animal models

Nine of these 14 GR mutations presented an elevated plasmatic level of the adrenocorticotropic hormone (ACTH) (Table 1), although also patients with normal ACTH levels were diagnosed (Table 1). Furthermore, patients carrying the Q501H mutation present a low level of ACTH despite adrenal hyperplasia. Thus, an increase of ACTH level might not be the only cause of adrenal gland hyperplasia in patients harbouring GR mutations. An autocrine positive regulatory feedback of glucocorticoid secretion was proposed that directly impacts NCI-H295R adrenocortical cell function [2]. Thus, following birth, the adrenal gland continues to mature undergoing rapid involution and differentiation into *zona fasciculata* (ZF) and *zona glomerulosa* mainly under the influence of ACTH and angiotensin II. At the age of 6 years, the *zona reticularis* will appear. Thus, GR may play a direct or indirect role in the adrenarche process. It could explain the adrenal gland hyperplasia diagnosed in a patient harbouring the E198X mutation only at the age of 12 and not at 3 years when the mutation has been found [64] (Figs. 1 and 3). Interestingly, 9 GR mutations out of 14 (64.3%) with adrenal hyperplasia are also associated with hypertension (Table 1), i.e. 47% of the GR mutations with hypertension are also associated with adrenal gland hyperplasia raising the question whether a direct link between hypertension and adrenal hyperplasia exists (Fig. 3). In several cases, an increase of the aldosterone level may explain hypertension. Furthermore, a high aldosterone level associated with an adrenal hyperplasia and hypokalaemia might be a sign of primary aldosteronism.

### Are GR mutations causative of primary aldosteronism?

Primary aldosteronism (PA) is the first and most common cause of secondary hypertension [35] characterized by high blood pressure, adrenal hyperplasia, hyperaldosteronism, low renin concentration and often hypokalaemia [6, 77] (Fig. 3). Often underdiagnosed, PA is prevalent in 5–10% of hypertensive patients and reaches more than 20% prevalence in patients suffering of resistant hypertension [6, 77]. Genetic somatic alterations were identified in aldosterone-producing tumours and adrenocortical carcinoma and linked to aberrant intracellular calcium signalling and altered ATPase function leading to enhanced *CYP11B*2 expression and increased aldosterone production [62, 77]. Patients with the familial form 3 of hyperaldosteronism presented with massive adrenal hyperplasia, severe hypokalaemia and high concentrations of the hybrid steroids 18-oxocortisol and 18-hydroxycortisol in urine [13].

It is important to note that PA might also be associated with hypercortisolism or subclinical Cushing’s syndrome. Cortisol-producing tumours were associated with aberrant cAMP-protein kinase A signalling. Furthermore, germline mutations in the tumour suppressor gene armadillo repeat containing 5 (ARMC5) were the underlying cause for primary bilateral macronodular adrenal hyperplasia (PBMAH) (see for review [29]). Indeed, recent studies demonstrated that the prevalence of hypercortisolism in PA is ranging from 12.1 to 77.6% [25]. Furthermore, an impairment of the glucose metabolism in PA is associated with cortisol co-secretion and may lead to diabetes type II underlining hypercortisolism as common clinical feature for PA [19]. PA patients with abnormal activation of glucocorticoid receptor in keratinocytes were reported [4].

In summary, due to the heterogeneity of the data collected, we cannot conclude that GR mutations are causative for PA. There is certainly additional need for evaluation of the renin-aldosterone ratio and the plasma renin activity (PRA). To further understand the underlying mechanisms of glucocorticoid resistance, adrenal gland hyperplasia and hypertension, animal models are useful to further dissect the development of the symptoms under normal and challenging conditions. In the following section, we will discuss the findings in transgenic rodents exhibiting GR mutations (Table 2).

**Table 2:** Overview of rodent models with altered GR expression and their phenotype

Nomenclature	Rodent model and study conditions	Adrenal hyperplasia	Corticosterone (Basal)	Blood Pressure	Ref
GR^NFLAsGR^	KO, constitutive Standard diet	Yes	Elevated	Not reported	[54]
GR^+/−^	Heterozygotes, constitutive Standard diet	Yes	Elevated	Not reported	[16]
GR^loxP;Tie−1−Cre+^	KO, vascular endothelium	Not reported	Not reported	Dexamethasone-induced hypertension	[66][22]
GR^loxP;Ksp−Cre+^	KO, distal nephron Dexamethasone (15 mg/L)	Not reported	Not reported	Less elevated following dexamethasone treatment	[21]
GR^βgeo/+^	Heterozygotes, constitutive Standard diet	Yes	Elevated	Elevated	[40]
Nr3c1^Pax8/LC1^	KO, kidney tubule- specific KO, High salt diet	Not reported	Not reported	Diastolic BP dipping increased following high salt	[8]
GR^+/em2^	Heterozygotes, High salt diet	Yes	Elevated	Elevated following high salt	[55][68]

*KO* knockout, *BP* blood pressure

### GR transgenic animal models and hypertension

The role of GR on salt sensitive hypertension, salt excretion and renal blood flow was first studied in GR haploinsufficient rodents. A lack of sensitivity to glucocorticoids was observed in mice carrying an impaired corticosteroid receptor function by inhibiting gene expression with antisense RNA. These mice showed increased plasma corticosterone as well as adrenocorticotropic hormone (ACTH) concentrations and slightly hyperplastic adrenal glands [54] (Table 2). Higher plasma corticosterone levels were also found in mice with loss of one functional GR allele (GR^+/−^) [16]. Further studies in these mice revealed the corticotropin-releasing hormone (CRH) as a major target for glucocorticoid feedback-control at the hypothalamic level [33]. Thereby, GR^+/−^ mice were lacking the GR-dependent regulation of the hypothalamus-pituitary axis (HPA) established during foetal development [58]. In models of globally reduced GR expression, salt-sensitivity and sustained hypertension were observed most likely reflecting sustained mineralocorticoid receptor activation [26, 55]. Mice in which the GR was disrupted by gene-trap integration of a β^geo^ reporter gene (GR^βgeo/+^, [39]) displayed a hyperactive HPA with elevated plasma corticosterone and larger adrenal glands due to hyperplasia as well as hypertension due to the activation of the renin–angiotensin–aldosterone system (RAAS) [39]. A high salt diet induced furthermore an increase of corticosterone excretion and the BP in these mice. Similarly, rats with GR haploinsufficiency (GR^+/em2^, [68]) when kept on a standard diet showed significantly increased plasma aldosterone and corticosterone levels and an adrenocortex hyperplasia but a normal systolic blood pressure. Salt-sensitive hypertension was only provoked in combination with high salt intake in the transgenic rats [68]. During prolonged salt exposure, both transgenic and control rats progressively reduced their salt intake [68]. While, in the mouse, an adaptive failure of the renal vasculature and tubule resulting in transient sodium retention was documented, a dysregulation of the soluble epoxide hydroxylase enzyme (sEH) that regulates omega 3 and omega 6 non-polysaturated fatty acid metabolism was reported in rat, with a significant increase in less active metabolites [68]. Further studies are needed to elucidate whether this is a cause or consequence of salt-sensitive hypertension and/or this may affect the endothelial function.

To better understand the role of the vasculature in the generation and maintenance of glucocorticoid-mediated hypertension, vascular smooth muscle-specific (GR^Tie−Cre^) knockout mice were analyzed [21]. These animals showed reduced vascular reactivity to dexamethasone, although their BP did not change significantly upon chronic oral administration of dexamethasone. Differences in circadian BP rhythm as well as higher baseline BP were observed suggesting that the vascular endothelial glucocorticoid receptor may function as a peripheral circadian clock [21]. Further studies are required to address the question whether glucocorticoid-mediated hypertension is primarily due to effects of glucocorticoids on the vasculature and not due to sodium retention and water absorption or both.

Persistent increased circulating glucocorticoids were proposed to contribute to nocturnal hypertension and induced a non-dipping blood pressure profile in mice with global reduction of GR abundance [28]. BP further increased by high salt diet in these mice, although systemic effects might not be excluded [26]. GR response elements (GREs) within the α-ENaC promoter region mediated glucocorticoid-induced transcription that might allow GR to contribute to the control of Na^+^ absorption [51]. Furthermore, GR activation dynamically regulated NCC phosphorylation and established the diurnal rhythm of NCC activity [27]. To further exclude systemic effects on BP by hypercortisolemia and understand the role of GR in combination with renal ion transporters in BP regulation, the implication of renal tubular GR was further analysed in mice lacking GR either along the nephron (Nr3c1^Pax8/LC1^ [8]) or only in the distal nephron [22]. Overall, Nr3c1^Pax8/LC1^ mice specifically lacking the renal tubular GR do not show alteration in sodium or potassium balance but present a transient sodium handling defect following diet switch to low sodium diet and an increased diastolic BP dipping upon switch to high salt diet. Analysis of Nr3c1^Pax8/LC1^ mice revealed that GR affected αENaC abundance and NCC activity in renal tubules [8]. Mice with a partial constitutive knockout in the distal nephron (GR^loxP;Ksp−Cre+^) exhibited mildly increased baseline BP, but similar hypertensive response to dexamethasone [22]. Interestingly, mice lacking the mineralocorticoid receptor (MR) along the nephron show increased GR protein abundance [8] and mice constitutively lacking the MR can partially be rescued with dexamethasone injections [3]. Mu and colleagues showed that dietary salt excess, coupled with β-adrenergic receptor stimulation, increased arterial BP via glucocorticoid receptors and WNK4 [42]. Salt loading increased blood pressure in isoproterenol-treated WT but not in distal nephron-specific glucocorticoid receptor knockout mice, suggesting that the GR is indispensable for βAR-mediated WNK4 downregulation and the development of salt-induced hypertension [18].

Glucocorticoid signalling regulates a wide range of systems in vertebrate organisms, and their responsiveness to glucocorticoids differs largely between individuals. Apart from the commonly used rodent models, the zebrafish has been proposed as an excellent in vivo model to study glucocorticoid resistance [61]. As a non-mammalian animal model, it has a GR β splice variant with dominant-negative activity and can thus be used to understand glucocorticoid action by GR β isoform overexpression or lacking glucocorticoid downregulation [61].

## Conclusion

Glucocorticoid signalling stimulates several pathways concurrently and converges on common mechanisms. Although a relatively high number of human subjects suffering from glucocorticoid resistance and carrying *NR3C1* mutations exhibit adrenal hyperplasia, this clinical feature may not be a prerequisite to develop hypertension. Nevertheless, not all patients with adrenal hyperplasia were analysed for GR mutations. Environmental factors as varying daily salt intake might play an additional important role here. In the contrary, there is a strong correlation between *NR3C1* gene mutations, salt-sensitive hypertension and high salt intake. Increased adrenocorticotropic steroids are known to promote renal sodium reabsorption and implicate both GR and mineralocorticoid receptor (MR) pathways that contribute to the pathogenesis of hypertension. We thus cannot exclude compensatory mechanisms and/or glucocorticoid-mediated mineralocorticoid activation. To dissect the underlying mechanisms, animal models are required that can be challenged and, hence, the development of hypertension can be followed over time. The use of inhibitors as sEH enzyme inhibitors in models with acute kidney injury (AKI), diabetic nephrology (DN), chronic kidney diseases (CKD), hypertension and other renal dysfunctions may present a potential therapy although this needs further experimental studies. More complete and standardized phenotyping of human subjects as well as adapted animal models will further help to dissect the underlying molecular mechanisms of adrenal gland hyperplasia and salt-sensitive hypertension.
